# Behavioral and Immunohistochemical Study of the Effects of Subchronic and Chronic Exposure to Glyphosate in Mice

**DOI:** 10.3389/fnbeh.2017.00146

**Published:** 2017-08-08

**Authors:** Yassine Ait Bali, Saadia Ba-Mhamed, Mohamed Bennis

**Affiliations:** Laboratory of Pharmacology, Neurobiology and Behavior (URAC-37), Faculty of Sciences Semlalia, Cadi Ayyad University Marrakech, Morocco

**Keywords:** glyphosate, locomotor activity, anxiety, depression, tyrosine-hydroxylase, serotonin, basolateral amygdala, medial prefrontal cortex

## Abstract

Many epidemiological studies have described an adolescent-related psychiatric illness and sensorimotor deficits after Glyphosate based herbicide (GBH) exposure. GBH exposure in animal models of various ages suggests that it may be neurotoxic and could impact brain development and subsequently, behavior in adulthood. However, its neurotoxic effects on adolescent brain remain unclear and the results are limited. The present study was conducted to evaluate the neurobehavioral effects of GBH following acute, subchronic (6 weeks) and chronic (12 weeks) exposure (250 or 500 mg/kg/day) in mice treated from juvenile age until adulthood. Mice were subjected to behavioral testing with the open field (OF), the elevated plus maze, the tail suspension and Splash tests (STs). Their behaviors related to exploratory activity, anxiety and depression-like were recorded. After completion of the behavioral testing, adult mice were sacrificed and the expression of tyrosine hydroxylase (TH) in the substantia nigra pars compacta (SNc) and serotonin (5-HT) in the dorsal raphe nucleus (DRN), the basolateral amygdala (BLA) and the ventral medial prefrontal cortex (mPFC) was evaluated using immunohistochemical procedure. Our results indicate that unlike acute exposure, both subchronic and chronic exposure to GBH induced a decrease in body weight gain and locomotor activity, and an increase of anxiety and depression-like behavior levels. In addition, the immunohistochemical findings showed that only the chronic treatment induced a reduction of TH-immunoreactivity. However, both subchronic and chronic exposure produced a reduction of 5-HT-immunoreactivity in the DRN, BLA and ventral mPFC. Taken together, our data suggest that exposure to GBH from juvenile age through adulthood in mice leads to neurobehavioral changes that stem from the impairment of neuronal developmental processes.

## Introduction

The widespread use of pesticides in various domains increases the risk of environmental contamination by different xenobiotics which can be potentially noxious for non-target organisms including human beings. The use of organophosphate (OP) Glyphosate (Gly) has been globally expanded. Indeed, around two-thirds of the total volume of Glyphosate-based herbicides (GBH) used has been delivered to the environment during the last decade (Myers et al., 2016), in step with the increased adoption of genetically engineered crops such as corn, soy and canola. This GBH is the active ingredient present in Roundup^®^ (Monsanto Company, St. Louis, MO, USA), currently the most heavily used herbicide worldwide (Powles et al., 1997).

Organophosphate (OP) exposure has been reported to be linked with mood disorders, such as depression and anxiety (Landrigan et al., 1999; London et al., 2005; Lee et al., 2007). Previous studies have demonstrated the potential risks of these pesticides, including their neurotoxic effects on developing organisms (Eaton et al., 2008). Furthermore, epidemiological studies have reported that long-term exposure to OPs elicits many neurological disturbances (Steenland et al., 1994; Roldan-Tapia et al., 2006). While classified under the herbicide class phosphonomethyl amino acids, Gly is often mischaracterized as an OP. This is likely due to the molecular structure being an organic molecule containing a phosphorus atom. However, clinical reports describing incidents of human ingestion of Gly do not reflect the classic symptoms for OP poisoning (salivation, lacrimation, urination, and defecation (Costa, 2008). Conversely, Seneff et al. (2015) highlighted the strong correlations between the increasing application of Gly in agriculture and the apparent surge of several neurological diseases at different ages. Abnormal EEG activity characterized by limb tremor (akinesia and rigidity) observed in Parkinsonian syndrome have been reported after professional exposure or accidental ingestion of a commercial mixture of Gly (Barbosa et al., 2001; Malhotra et al., 2010; Wang et al., 2011). In addition, data derived from structural MRI studies highlighted in a subject exposed to Gly changes in the T2 signal in substantia nigra, periaqueductal gray and globus pallidus, revealing likely lesions in these structures (Barbosa et al., 2001). Moreover, in human studies, Gly has been detected in the brain and cerebro-spinal fluid following commercial mixtures exposure, attesting that the active component can traverse the blood brain barrier (Menkes et al., 1991; Sato et al., 2011).

Research on animal models confirmed the neurotoxic effects of Gly. Indeed, Negga et al. (2012) revealed that exposure to GBH cause neuronal death, especially of GABAergic and dopaminergic neurons in the nematode *Caenorhabditis elegans*. It was also reported that Gly induces acetylcholinesterase activity alteration in the brain and the muscle of fishes exposed either to pure Gly or Roundup^®^ (Modesto and Martinez, 2010; Menéndez-Helman et al., 2012; Sandrini et al., 2013; Samanta et al., 2014). An increasing body of literature revealed that Gly was able to provoke oxidative stress in specific rat brain regions such as substantia nigra, cerebral cortex and hippocampus (Astiz et al., 2009; Cattani et al., 2014).

In addition, the exposure to the GBH Roundup^®^ during pregnancy and lactation provokes decrease in glutamate uptake and metabolism within glial cells, and increasing glutamate release in the synaptic cleft in the hippocampus of orally exposed rat’s offspring, leading to glutamate excitotoxicity (Gallegos et al., 2016).

Apart from the prenatal or postnatal period, the adolescence age is also a critical phase of brain development (Van Waes et al., 2011). Indeed, adolescence is a critical ontogenic period characterized by the maturation of brain processes that underlie higher cognitive functions, social and emotional behaviors (Spear, 2000). This period is often one of increased vulnerability and adjustment (Steinberg, 2005). In humans and rodents, adolescence is a time of extensive neural reorganization/pruning, in which long-term deleterious effects due to drug and environmental factor exposures are greater than at later times in life (Spear, 2000; Mendola et al., 2002).

The evident structural and neurochemical changes during adolescence are characterized by increases in functional connectivity between regions of the prefrontal cortex and several areas of the limbic system, especially the dopaminergic and serotoninergic systems, concomitant with changes in emotional and cognitive function regulation (Wahlstrom et al., 2010; Naneix et al., 2012; Spear, 2013). Moreover, during adolescence, brain areas continue to develop, such as the amygdala, hippocampus, and prefrontal cortex (Giedd and Rapoport, 2010). Thus, disturbing serotoninergic and dopaminergic systems in the aforementioned brain regions may result in permanent behavioral alterations associated with motor and emotional functions. In light of this, an important determinant that remains to be investigated is whether GBH exposure, following exposure to a Gly-containing herbicide, acts directly on the adolescent and adult brain resulting in subsequent sensorimotor and emotional function controlled by serotoninergic and dopaminergic systems.

## Materials and Methods

### Animals

Male Swiss mice (1 month old) were obtained from the animal husbandry of the Faculty of Sciences, Cadi Ayyad University, Marrakech, Morocco. The choice of the use of males in this study was based on the fact that several studies have reported a difference in sensitivity of the brain to the toxicity to several substances linked either to a sex difference in the permeability of the cerebrospinal blood barrier (Kato, 1974), or to increased susceptibility to toxins in females with respect to males (Sonawane and Baksi, 1980; Rhodes and Rubin, 1999; Kim et al., 2005). The animals were housed in Plexiglas cages (6 mice/cage; 30 cm × 15 cm × 12 cm) under standard conditions of temperature (22 ± 2°C) and photoperiod 12 h/12 h (lights on at 08:00 h). Food and water were available *ad libitum*. All procedures were conducted in accordance with approved institutional protocols, and with the provisions for animal care and use prescribed in the scientific procedures on living animals, European Council Directive: EU2010/63. All efforts were made to minimize any animal suffering. The study was approved by the Council Committee of Research Laboratories of the Faculty of Sciences, Cadi Ayyad University, Marrakech.

### Pesticide

Roundup^®^ herbicide (Gly concentration 360 g/l in the form of Gly isopropylamine salt 486 g/l) with molecular formula C_6_H_17_N_2_O_5_P, molecular weight of 228.183 g/Mol, melting point 200°C and density 1.218 g/cm3 was used in the liquid commercial form supplied by Monsanto Company (St. Louis, MO, USA).

### Acute, Subchronic and Chronic Toxicity Assessment of GBH

#### Study Design

**Figure 1:**
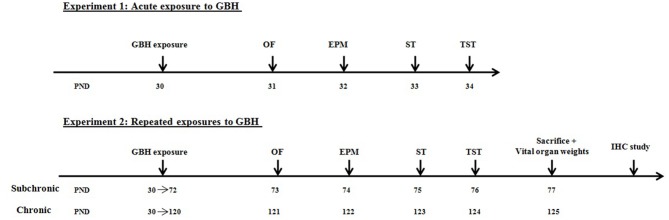
Experimental design of the acute and repeated exposures to glyphosate based herbicide (GBH) in male mice.

Healthy mice were assigned to three experimental groups (acute, subchronic and chronic: *n* = 18/group), and each group was subjected either to orally gavages by NaCl 0.9% (control *n* = 6), by 250 mg (*n* = 6) or 500 mg/kg/day (*n* = 6) of GBH. These doses were selected on the basis of Gly no-observed adverse effect level (NOAEL) of 500 mg/kg/day for subchronic and chronic toxicity (EPA, 1993). Gly solutions were prepared daily to minimize the risk of degradation since Gly half-life in water varies from 49 days to 70 days (Mercurio et al., 2014). The mice assigned to the acute group received one administration of GBH while the subchronic and chronic groups were treated daily for 6 and 12 weeks, respectively. All mice were observed and their body weights were measured daily during the exposure period. Following the end of treatment, all groups of mice were tested for their locomotor activity and affective behavior on consecutive days. On the last day of the experiment, only the subchronic and chronic treated animals were sacrificed to perform vital organs weight analysis and immunochemistry study (Figure 1).

#### Effects of GBH Exposure on Mouse Behavior

All treated animals were tested between 9:00 h and 12:00 h in the light cycle. Before behavioral testing, the animals were gently handled and they were individually familiarized with the testing room and the test arena for 5 min prior to the test circumstance. To minimize subjectivity, the behavior of mice was recorded and analyzed using Ethovision XT Noldus 8.5 video tracking program (Noldus Information Technology b.v., Wageningen, Netherlands), connected to a video camera (JVC). The video camera was positioned 2.5 m above the arena, inside the vertical projection of a wall, covering the entire view of the arena. Tracking of the animal was based on contrast relative to the background. Two tracking points were specified: on the head and the center of gravity of the animal.

##### Open field test

The Open field (OF) test is the one of the commonly used test to assess locomotor activity and emotional reactivity in rodents placed into novel environment (Wilson et al., 1976). The Activity monitoring was carried out in black Plexiglas arena measuring 50 × 50 × 50 cm, equipped with the video-based Ethovision System (Noldus, Wageningen, Netherlands). The OF apparatus was illuminated by a 75W lamp placed in porthole diffusing light and located at 200 cm from the device, allowing the center of the arena to be under a dim light (100 lx). Mice were placed individually into the arena and locomotor activity was assessed for 20 min using the video-tracking system. Parameters recorded were total distance moved, velocity, and the percentage of the time spent in the arena center (15 × 15 cm). The test box was cleaned with 70% ethanol between each test.

##### Elevated-plus-maze test

The elevated-plus-maze (EPM) is the widely paradigm used to assess anxiety-like behavior in rodents (Handley and Mithani, 1984; Lapiz-Bluhm et al., 2008). The EPM is comprised of two opposing open arms (50 × 5 cm) and two closed arms (50 × 5 × 15 cm), which are joined at a square central area (5 × 5 cm) to form a plus sign. The maze floor and the side/end walls (15 cm height) of the enclosed arms were made of clear Plexiglas. The entire apparatus was elevated to a height of 45 cm above the floor. The EPM apparatus was illuminated by a 75W lamp, located at 200 cm from the device, allowing an approximate brightness of 200 lux. For testing, the mouse was removed gently from its home cage and placed in the central arena of the EPM, facing the junction of an open and closed arm. The mouse was allowed to freely explore the maze for 5 min, while its behavior was recorded for offline analysis. The time spent in the open arm over the total time spent in both arms, the number of entries to the open arm vs. the total number of entries corresponding to the ratio of time spent and the ratio of open arm entries, respectively, and the anxiety index, which is 1 − (ratio time + ratio entry)/2, were quantified during the test session.

##### Tail suspension test

The Tail Suspension test (TST) is one of the common behavioral tests adopted to assess the potential antidepressant-like effects in rodents (Cryan and Holmes, 2005). Each individual mouse was suspended by the tail with adhesive tape above the ground, approximately 40 cm from the floor. The course of the TST over a single 6 min session was scored. The immobility time during the last 4 min of a session was recorded.

##### Splash test

The Splash test (ST) is widely used to evaluate the motivational deficits and self-care difficulties as symptoms of depression in rodents (David et al., 2009; Amiri et al., 2015). In this test, the decrease of grooming behavior of mice (considered as an index of self-care and set of emotional and behavioral alterations, including persistent depressed mood and loss of interest or pleasure as core symptoms: Vural et al., 2007) was measured. Briefly, a 10% sucrose solution was squirted on the dorsal coat of animals in their home cage and the time spent grooming was recorded for a period of 5 min after the sucrose application.

#### Tissue Preparation

Upon completion of behavioral testing, subchronic and chronic treated mice were anesthetized with an intraperitoneal injection of urethane 40% (1 g/kg, from Sigma–Aldrich, France) and transcardially perfused with saline solution (0.9%), followed by 4% paraformaldehyde in phosphate buffered saline (PBS; 0.1 M). The brain, liver, kidneys and lung were removed and weighed. The relative weight was calculated as organ weight/body weight. The brains were removed, post-fixed in the same fixative for 12 h and cryoprotected overnight in 30% sucrose. They were then cut on a freezing microtome (Leica Microsystems, Germany) into 40 μm frontal sections containing substantia nigra pars compacta (SNc), the dorsal raphe nucleus (DRN), the ventral part of the medial prefrontal cortex (mPFC) and the basolateral nucleus of the amygdala (BLA). These regions of interest were determined according to stereotaxic atlas of Paxinos and Franklin (2001).

#### Immunochemistry

Slides containing the region of interest were incubated with hydrogen peroxide at 1% for 30 min to block endogenous peroxidase activity, and washed five times for 10 min in PBS solution. Then they were incubated for 60–90 min in PBS solution containing bovine serum albumin-containing (BSA; SERVA ELECTROPHORESIS GmbH, Germany) and 1% Triton X-100 (ACROS ORGANICS, USA) at 0.3% (PBS-T-BSA), under stirring at room temperature. Thereafter, the sections were incubated with anti-tyrosine hydroxylase (TH) polyclonal primary antibody produced in rabbit (SIGMA ALDREICH GmbH, Germany) or with an anti-serotonin (5-HT) polyclonal antibody produced in rabbit (CALBIOCHEM, mAb (GA-5) IF03L, Germany) diluted 1:1000 and 1:5000, respectively, in PBS-T-BSA overnight, under stirring at 4°C. Sections were washed five times in PBS for 10 min each, and then incubated with rabbit biotinylated secondary antibody diluted 1:400 in PBS-T under stirring overnight at 4°C, and rinsed five times in TBS for 10 min each. Sections were then rinsed in PBS, and incubated for 1 h 30 at room temperature in avidin-biotin-peroxidase complex (Vectastain Elite ABC Kit, Vector Laboratories, Burlingame, CA, USA) diluted at 1:200 in PBS. After rinsing in PBS, the peroxidase activity was visualized with 3-3′-diaminobenzidine tetrahydrochloride (SIGMA ALDREICH GmbH, Germany) at 0.025% in Tris buffer (Pre-set) 0.05 M, (PROCHILAB, France) containing hydrogen peroxide (0.006%). Processing was stopped by rinsing sections for 3 × 10 min in PBS. Sections were mounted on gelatine-coated slides, dehydrated in graded series of ethanol, cleared in xylene and cover-slipped with Eukitt, and examined under a light microscope for the quantification of TH or 5-HT immunoreactivity.

#### Acquisition, Image Processing and Quantification of Immunoreactive Neurons

The images of brain sections were acquired at high magnification (×100 and ×400) on an Olympus BH-2 microscope equipped with a camera Olympus DP71. The reconstruction of a complete photomicrograph association of different shots and their treatment was made through image processing software Adobe Photoshop^®^. For each brain region, four representative sections from anterior to posterior were chosen and counted to minimize variability. Individual means were obtained by quantification of the intensity of immunostaining for TH and 5-HT bilaterally in four sections from each region, by an experimenter blind to the treatment conditions, using ImageJ software. Sections were chosen by correspondence to the Paxinos and Franklin (2001) stereotaxic atlas. TH densitometry was quantified in SNc (Interaural = 0.88 mm, Bregma = −2.92 mm), 5-HT immunoreactive cells were quantified in the DRN (rostral part: Interaural = −0.80 mm, Bregma = −4.60 mm; caudal part: Interaural = −1.04 mm, Bregma = −4.84 mm), the ventral mPFC (Interaural = 5.78 mm, Bregma = 1.98 mm) and the BLA (Interaural = 1.98 mm, Bregma = −1.82 mm).

#### Statistical Analysis

To compare data from behavioral testing with immunohistochemical findings between the treated groups and control, a statistical analysis of these different independent variables was performed by two way ANOVA (treatment and the period of treatment), using the Sigma Plot software 11.0. Data from the body and organ weight were analyzed using two way ANOVA with treatment as the factor. *Post hoc* analysis was performed using Holm-Sidak *post hoc* test. The distribution of the data related to each measure was assessed automatically for their normality by the software before performing any kind of statistical analysis. Results are presented as mean ± standard error of the mean (SEM). The significance threshold was set at *p* < 0.05.

## Results

### GBH Effect on Body Weight Gain

Body weight gain was not affected in mice after acute exposure to the two GBH concentrations tested on day 1 (*F*_(2.17)_ = 0.46; *p* = 0.64) and on day 7 (*F*_(2.17)_ = 0.06; *p* = 0.94). However, subchronic and chronic exposure at doses of either 250 mg/kg or 500 mg/kg of GBH, resulted in reduced body weight gain of mice over the 15-day experimental period. On day 30, the treated animals showed a slight recovery of their body weight that remained statistically significant compared with controls (Table 1).

**Table 1:** Subchronic and chronic effect of Glyphosate based herbicide (GBH) on body weight gain (g).

Days	Subchronic treatment			ANOVA *F*_(2.17)_	Days	Chronic treatment			ANOVA *F*_(2.17)_
	**Control**	**250 mg/kg**	**500 mg/kg**			**Control**	**250 mg/kg**	**500 mg/kg**	
Day 1	0.98 ± 0.28	0.85 ± 0.12	0.78 ± 0.18	0.23	Day 1	0.80 ± 0.24	0.63 ± 0.09	0.51 ± 0.1	0.74
Day 7	2.30 ± 0.43	−0.83 ± 0.14^###^	−1.31 ± 0.16^###^	48.90***	Day 7	1.75 ± 0.25	−1.45 ± 0.54^###^	−1.58 ± 0.51^###^	17.1***
Day 15	2.95 ± 0.45	0.36 ± 0.24^###^	−0.13 ± 0.38^###^	20.03***	Day 15	3.48 ± 0.71	0.18 ± 0.39^###^	−0.88 ± 0.37^###^	19.23***
Day 30	4.16 ± 0.46	2.60 ± 0.15^###^	0.60 ± 0.21^###$$$^	42.06***	Day 30	4.65 ± 1.03	1.10 ± 0.61^##^	0.73 ± 0.33^###^	10.34**
Day 40	5.53 ± 0.35	2.98 ± 0.19^###^	1.18 ± 0.23^###$$$^	60.81***	Day 40	5.79 ± 0.96	2.23 ± 0.34^###^	0.78 ± 0.2^###$^	19.79***
					Day 60	10.25 ± 0.88	5.06 ± 0.85^###^	4.58 ± 0.44^###^	17.28***
					Day 90	12.88 ± 0.79	8.68 ± 0.95^##^	8.27 ± 0.56^###^	10.37**

Results are presented as mean ± SEM. **P < 0.01, ***P < 0.001: ANOVA analysis. ^##^*P* < 0.01, ^###^*P* < 0.001, ^$^*P* < 0.05, ^$$$^*P* < 0.001: the *post hoc* analysis. The “#” comparison to the control. The “$” compared to the 250 mg.

### GBH Effect on Organ Weight

The organ relative weights of both treated and control groups are presented in Table 2. Two-way ANOVA analysis showed a significant difference in the weight of brain, liver, kidneys and lung among the factor of treatment (*F*_(2.17)_ = 13.51, *p* < 0.001; *F*_(2.17)_ = 12.13, *p* < 0.001; *F*_(2.17)_ = 10.76, *p* < 0.001; *F*_(2.17)_ = 7.97, *p* < 0.001, respectively), and only in the brain relative weight for the period factor (*F*_(2.17)_ = 39.69, *p* < 0.001), as well as the interaction of treatment × period for brain and liver relative weights (*F*_(2.17)_ = 11.70, *p* < 0.001; *F*_(2.17)_ = 5.15, *p* < 0.05). The *post hoc* analysis confirmed that treated groups, especially the 500 mg/kg group, exhibited significant decrease in organ’s relative weight following GBH-exposure (*p* < 0.05; Table 2).

**Table 2:** Effect of GBH on relative organ weight.

Organs	Subchronic treatment			Chronic treatment		
	**Control**	**250 mg/kg**	**500 mg/kg**	**Control**	**250 mg/kg**	**500 mg/kg**
Brain	0.03 ± 0.002	0.03 ± 0.002	0.02 ± 0.001^###$$$^	0.02 ± 0.009	0.02 ± 0.009	0.01 ± 0.004^###^
Kidneys	0.01 ± 0.001	0.01 ± 0.001	0.01 ± 0.009	0.02 ± 0.007	0.01 ± 0.001^##^	0.01 ± 0.005^###^
Liver	0.07 ± 0.002	0.08 ± 0.01	0.04 ± 0.004^##$$^	0.09 ± 0.002	0.05 ± 0.005^###^	0.05 ± 0.004^###^
Lung	0.02 ± 0.002	0.02 ± 0.001	0.01 ± 0.002	0.02 ± 0.007	0.01 ± 0.002^##^	0.01 ± 0.001^##^

*Results are presented as mean ± SEM. ^#^*p* < 0.05, ^##^*p* < 0.01, ^###^*P* < 0.001, ^$$^*P* < 0.01, ^$$$^*p* < 0.001: the *post hoc* analysis. The “#” comparison to the control. The “$” comparison to the 250 mg*.

### Behavioral Changes after GBH Exposure

#### Open Field Test

**Figure 2:**
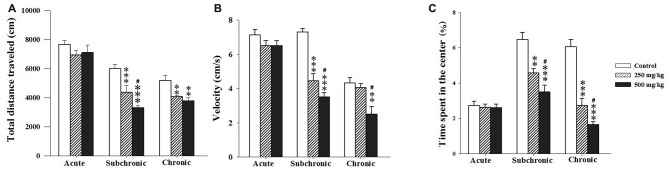
Effect of the acute and repeated exposures to GBH on locomotor activity. **(A)** Total distance traveled. **(B)** The moving velocity. **(C)** The time spent in the center. Results are presented as mean ± standard error of the mean (SEM). **P < 0.01; ***P < 0.001. The *** refers to the control vs. 250 mg/kg and 500 mg/kg group comparison and the “#” refers to the 250 mg/kg vs. 500 mg/kg group comparison. ^#^*P* < 0.05: the *post hoc* analysis.

As indicators of locomotor activity and anxiety-like levels, we registered the total distance traveled over the maze (activity) and the time spent in the central area. Two-way ANOVA analysis of the total distance traveled, the velocity and the percentage of the time spent in the central zone revealed significant differences among the factors of treatment (*F*_(2.17)_ = 18.93, *p* < 0.001; *F*_(2.17)_ = 3.06, *p* < 0.001; *F*_(2.17)_ = 51.18, *p* < 0.001, respectively) and period (*F*_(2.17)_ = 76.21, *p* < 0.001; *F*_(2.17)_ = 71.16, *p* < 0.001; *F*_(2.17)_ = 38.04, *p* < 0.001 respectively) as well as an interaction of treatment × period for velocity and the percentage of time spent in the central zone (*F*_(2.17)_ = 11.02, *p* < 0.001; *F*_(2.17)_ = 13.22, *p* < 0.001). The *post hoc* analysis using Holm-Sidak test confirmed that treated groups, especially the 500 mg/kg group, exhibited significant decrease in all parameters recorded during the OF test following subchronic and chronic GBH-exposure (*p* < 0.05; Figures 2A–C).

#### Elevated-Plus-Maze Test

**Figure 3:**
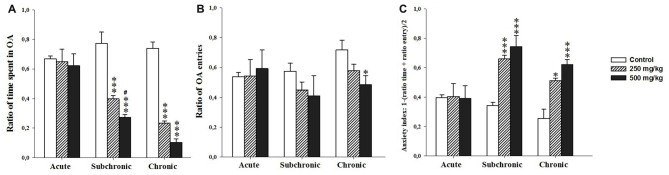
Effect of the acute and repeated exposures to GBH on anxiety-like behavior. **(A)** The ratio of time spent in open arm. **(B)** The ratio of open arm entries. ** (C)** The anxiety index. Ratio = time spent in each arm/(time spent in open arm + time spent in closed arm). Results are presented as mean ± SEM. *P < 0.05; ***P < 0.001. The *** refers to the control vs. 250 mg/kg and 500 mg/kg group comparison and the “#” refers to the 250 mg/kg vs. 500 mg/kg group comparison. ^#^*P* < 0.05: the *post hoc* analysis.

Two-way ANOVA test demonstrated significant difference in ratio of time spent in the OA and the anxiety index recorded during the EPM among the factors treatment (*F*_(2.17)_ = 1.57, *p* < 0.001; *F*_(2.17)_ = 17.75, *p* < 0.001 respectively) and period (*F*_(2.17)_ = 24.35, *p* < 0.001; *F*_(2.17)_ = 8.77, *p* < 0.001, respectively) as well as the interaction of treatment × period (*F*_(2.17)_ = 10.48, *p* < 0.001; *F*_(2.17)_ = 4.53, *p* < 0.001, respectively). However, the number of open arm entries showed no significant difference following GBH exposure (treatment (*F*_(2.17)_ = 1.57, *p* > 0.05) and period (*F*_(2.17)_ = 1.55, *p* > 0.05)). There was no interaction for treatment × period (*F*_(2.17)_ = 0.83, *p* > 0.05). The *post hoc* comparisons confirmed that the treated groups, especially 500 mg/kg group, showed a significant decrease in the time spent in the open arm (*p* < 0.001) and increase in the anxiety index (*p* < 0.05) with respect to control (Figures 3A–C).

#### Tail Suspension and Splash Tests

**Figure 4:**
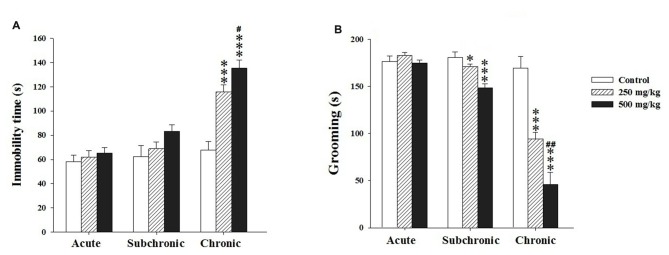
Effect of the acute and repeated exposures to GBH on depression-like behavior. **(A)** The immobility time in the tail suspension test (TST). **(B)** The grooming time in the splash test (ST). Results are presented as mean ± SEM. *P < 0.05; ***P < 0.001. The *** refers to the control vs. 250 mg/kg and 500 mg/kg group comparison and the “#” refers to the 250 mg/kg vs. 500 mg/kg group comparison. ^#^*P* < 0.05, ^##^*P* < 0.01: the *post hoc* analysis.

Two way ANOVA analysis of the immobility and grooming time revealed a significant differences among the factors treatment (*F*_(2.17)_ = 20.12, *p* < 0.001; *F*_(2.17)_ = 39.39, *p* < 0.001, respectively) and period (*F*_(2.17)_ = 42.02, *p* < 0.001; *F*_(2.17)_ = 92.41, *p* < 0.001, respectively) as well as in the interaction treatment × period (*F*_(2.17)_ = 7.31, *p* < 0.001; *F*_(2.17)_ = 19.89, *p* < 0.001, respectively). The *post hoc* comparisons using Holm-Sidak test confirmed that the treated groups showed a significant increase in the immobility time only following chronic treatment (*p* < 0.001) and a decrease in grooming time (*p* < 0.05) after both subchronic and chronic exposure (Figures 4A,B).

### Immunochemistry

#### TH-Immunoreactivity

**Figure 5:**
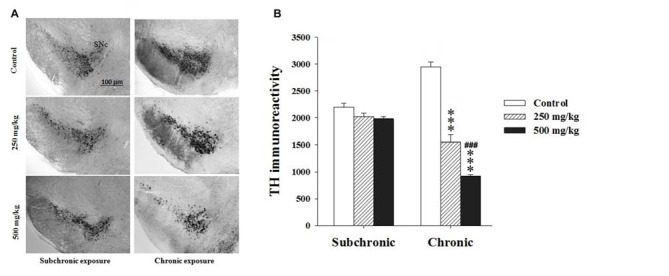
Effect of subchronic and chronic exposure to GBH on dopaminergic neurons. **(A)** Photomicrographs of mice brain cross sections showing the tyrosine hydroxylase (TH)-immunoreactive neurons at the SNc. **(B)** The intensity of TH Immunoreactivity at the SNc in control and treated mice. SNc, substantia nigra pars compacta. Results are presented as mean ± SEM. ***P < 0.001. The *** refers to the control vs. 250 mg/kg and 500 mg/kg group comparison and the “#” refers to the 250 mg/kg vs. 500 mg/kg group comparison. ^###^*P* < 0.001: the *post hoc* analysis.

Using TH, a key enzyme involved in dopamine synthesis, two-way ANOVA analysis of the TH immunoreactivity (TH^+^) revealed significant difference among the factors treatment (*F*_(2.17)_ = 107.41, *p* < 0.001) and period (*F*_(2.17)_ = 16.39, *p* < 0.001) as well as in the interaction of treatment × period (*F*_(2.17)_ = 69.04, *p* < 0.001). The *post hoc* comparisons confirmed that the treated groups showed a significant decrease in TH^+^ only following chronic treatment (*p* < 0.001; Figure 5).

#### Serotonin Immunoreactivity

**Figure 6:**
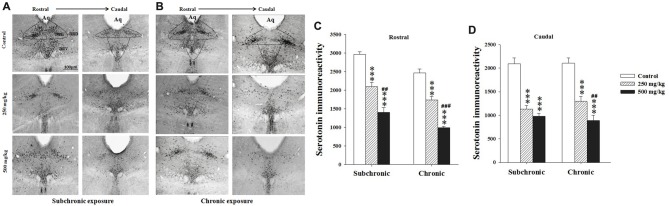
Effect of subchronic and chronic exposure to GBH on serotoninergic neurons. **(A)** Photomicrographs of mice brain cross sections showing the serotonin (5-HT)-immunoreactive neurons across the rostral to the caudal extent of the dorsal raphe nucleus (DRN) after subchronic exposure. **(B)** The intensity of 5-HT immunoreactivity at the DRN in control and treated mice following chronic exposure. **(C,D)** The intensity of 5-HT immunoreactivity at the DRN in control and treated mice. Aq, Cerebral aqueduct. DRD, dorsal raphe dorsal (dorsomedial), DRV, dorsal raphe ventral (ventromedial). Results are presented as mean ± SEM. ***P < 0.001. The *** refers to the control vs. 250 mg/kg and 500 mg/kg group comparison and the “#” refers to the 250 mg/kg vs. 500 mg/kg group comparison. ^##^*P* < 0.01, ^###^*P* < 0.001: the *post hoc* analysis.

**Figure 7:**
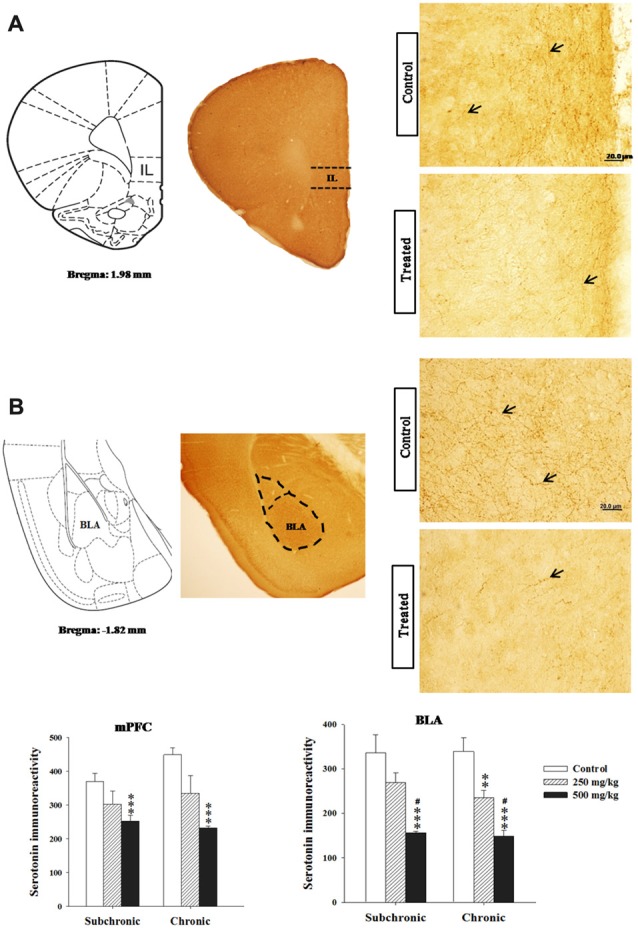
Effect of subchronic and chronic exposure to GBH on serotoninergic fibers. **(A)** Photomicrographs of mice brain cross sections showing the 5-HT-immunoreactive fibers and the intensity of 5-HT immunoreactivity in the mPFC. **(B)** Photomicrographs of mice brain cross sections showing the 5-HT-immunoreactive fibers and the intensity of 5-HT immunoreactivity in the basolateral nucleus of the amygdala. mPFC, ventral medial prefrontal cortex; BLA, basolateral nucleus of amygdale; IL, the infralimbic cortex corresponding to the ventral mPFC. The arrows refer to 5-HT fibers. Results are presented as mean ± SEM. **P < 0.01; ***P < 0.00. The *** refers to the control vs. 250 mg/kg and 500 mg/kg group comparison and the “#” refers to the 250 mg/kg vs. 500 mg/kg group comparison. ^#^*P* < 0.05: the *post hoc* analysis.

Two way ANOVA analysis of 5-HT immunoreactivity in the rostral and caudal parts of the DRN, the mPFC and basolateral amygdala (BLA) nucleus revealed significant differences for period in all regions studied (*F*_(2.17)_ = 122.24, *p* < 0.001; *F*_(2.17)_ = 73.41, *p* < 0.001; *F*_(2.17)_ = 15.32, *p* < 0.001; *F*_(2.17)_ = 28.98, *p* < 0.001, respectively), and in the rostral part of the DRN (*F*_(2.17)_ = 28.16, *p* < 0.001; Figures 6, 7). There was no significant difference in the interaction between treatment × period (*p* > 0.05). The *post hoc* comparison confirmed that the treated groups, especially 500 mg/kg group, showed a significant decrease in 5-HT densitometry in all structures studied following both subchronic and chronic treatment in respect to the control (*p* < 0.05; Figures 6, 7).

## Discussion

Because of the scarce information on the neurobehavioral effects of GBH in mammals, the present research was designed to gain insights concerning the effects of acute or repeated exposures to GBH on developing brain of juvenile and adult mice.

Our results showed that subchronic and chronic exposure to GBH in mice induced a significant decline in body weight gain for both treated groups. These results are in agreement with those obtained using comparable doses in adult mice (Jasper et al., 2012) and in rabbits (Yousef et al., 1995). According to Chahoud et al. (1999), weight loss is the main important indicator of toxicity, which may be associated with Roundup^®^’s capacity to provoke reactive oxygen species production. In addition, the decrease in the food consumption and the anorexic effect of Gly could lead to lower relative organs’ weight (ROW; Beuret et al., 2005). Thus, the assessment of ROW clearly revealed that GBH exposure induced a significant diminution of brain and liver weights after 6 weeks of treatment, while it affected brain, kidneys, liver and lung’s weight following 12 weeks of treatment. Several studies showed that Gly residues in the kidney, liver and lung were comparable to those found in the urine. This means that the Gly does not pass through the urine without affecting the organism (Krüger et al., 2014; Seneff et al., 2015). These results are consistent with previous studies in F344/N rats and B6C3F1 mice (World Health Organisation (WHO), United Nations Environment Programme, the International Labour Organisation, 1994). Similar results have been found in animals exposed to other pesticides such as deltamethrin, fenvalerate and diazinon (Kalender et al., 2006; Kilian et al., 2007). Indeed, in the same studies, the decrease of body weight gain recorded was due to malabsorption of nutrients induced by the gastro-intestinal tract impairment or inhibition of protein synthesis.

Acute exposure to GBH showed no change in locomotor activity. In contrast, subchronic and chronic treated animals showed a hypoactive profile. Our results are similar to those obtained by Hernández-Plata et al. (2015), who showed that repeated intraperitoneal injections of 100 or 150 mg/kg of Gly causes hypoactivity in adult male rats and those of Gallegos et al. (2016) in prenatally treated rats. Likewise, rats treated with methylparathion OP (Sun et al., 2006), diisopropylfluorophosphate pesticide (Bushnell et al., 1991) or dichlorvos-exposed (Binukumar et al., 2010) exhibited decreases in locomotor activity. Corroborating these findings, several OPs have been linked to movement and coordination deficits in humans following occupational exposures (Lotti and Moretto, 2005; Ehrich and Jortner, 2010). Further, many studies have reported that the locomotor activity reduction is positively correlated with the loss of dopaminergic neurons in the substantia nigra, as well as DA receptors (Bano et al., 2014; Gallo et al., 2015). Indeed, both the nigrostriatal and mesolimbic dopaminergic systems are implicated in the control of motor and motivated behaviors, and they have been shown to be susceptible to herbicides. For example, exposure to pesticides has frequently been defined as a major contributor to the risk of PD development (Hatcher et al., 2008).

During adolescence, Peak levels of extracellular dopamine are observed (Philpot et al., 2009) and that firing of dopamine cells and neurotransmitter turnover in target regions is higher in adolescents than adults (Tarazi et al., 1998; Moll et al., 2000; Placzek et al., 2009; McCutcheon et al., 2012). Moreover, increased studies reported the vulnerability of the nigrostriatal dopaminergic system following exposure to pesticides during critical periods of neurodevelopment, thus compromising the integrity and viability of this system (Cory-Slechta et al., 2005a,b). In light of this, the alterations of the dopamine system seen in our study coupled with locomotor hypoactivity are particularly interesting, when compared with previous work that demonstrated a reduction in the TH levels that paralleled motor activity deficiency after paraquat and maneb pesticide (Thiruchelvam et al., 2000). In agreement with our results, the exposure to Gly-containing pesticide leads to degeneration of GABA and dopamine neurons in *Caenorhabditis elegans* (Negga et al., 2012) and induces loss of cardiolipin content and mitochondrial transmembrane potential, especially in SNc, with a concomitant increase in fatty acid peroxidation (Astiz et al., 2009). According to Jackson et al. (2002) and Ricci et al. (2003), mitochondrial transmembrane potential loss reflects an early stage of apoptosis and release of many mitochondrial proteins into the cytosol during the first steps of programmed cell death. Additionally, Gui et al. (2012) reported a cell death in the dopaminergic cell line PC12 by Gly through apoptotic and autophagic mechanisms. Recently, Hernández-Plata et al. (2015) showed a reduction of the D1-DA receptor binding in the nucleus accumbens, accompanied by a significant decrease in ambulatory activity observed after Gly administration. Furthermore, following only 150 mg/kg of Gly administration, it was reported that striatal extracellular DA levels decreased by 50% in the microdialysis sample (Hernández-Plata et al., 2015).

On the other hand, the results revealed that subchronic and chronic exposure to the GBH induced an anxiogenic-like behavior. Similarly to our data, Sánchez-Amate et al. (2001) and Chen et al. (2011) found that repeated exposure of rats to another OP chlorpyrifos induces anxiogenic-like behavior. Conversely, Gallegos et al. (2016) observed that prenatal exposure to 100 or 200 mg/kg of GBH produced an anxiolytic-like effect in rats. These differential results may probably due to the different exposure periods. In fact, our study focused mainly on juvenile and adult age, whereas Gallegos et al. (2016) paid more attention to the prenatal exposure period.

Moreover, our results revealed that GBH exposed mice showed significant depression-like behavior. Our findings were in agreement with Chen et al. (2014) who showed that adolescent rats exposed to chlorpyrifos exhibited depression-like phenotype. Moreover, this result shows similarities to those of other OPs (Aldridge et al., 2004; Roegge et al., 2008). Indeed, previous researches that investigated the effects of methamidophos and diazinon at neonatal age and adulthood detected behavioral changes associated with depression (Lima et al., 2009) and changes in neurochemical markers of 5-HT function (Lima et al., 2011).

It is well established that several neuronal systems, in particular the serotoninergic system innervating cortical and limbic brain structures, are involved in the pathophysiology of anxiety and depression related behaviors (Azmitia and Segal, 1978; Vertes, 1991). Indeed, it has been shown that 5-HT system sends anatomical connections to the amygdala (Bobillier et al., 1976; Sadikot and Parent, 1990) and the ventral mPFC (Steinbusch, 1981; O’Hearn and Molliver, 1984) as pivotal structures contributing to emotional disturbances. Moreover, besides the changes in 5-HT transporters and their receptor activity, there is an increase in the release of 5-HT from the DRN in adolescence in comparison with adult age (de Jong et al., 2006), and the levels of 5-HT in several brain areas are also increased. Thus, The alteration of serotoninergic homeostasis during its development may result in a pattern of modified brain connections, leading to the loss of synapses and dendritic arborization, which normally occurs from puberty to adulthood (Barros et al., 2006; Zhang et al., 2013) and permanent behavioral alterations may be induced in adults (Whitaker-Azmitia, 2005; Ansorge et al., 2008).

Furthermore, the ventral mPFC is of special interest because it is strongly implicated in the expression of behavioral and autonomic responses to emotionally relevant stimuli, and imaging studies highlight abnormalities in the structure and function of this region in patients with mood disorder (Kennedy et al., 2001; Drevets et al., 2008). In agreement with these findings, our immunohistochemical data have shown a decrease of immunoreactive-neurons of the DRN and serotoninergic fibers in the BLA and in the ventral mPFC of mice following subchronic and chronic GBH exposure. Our results joined those obtained by Anadón et al. (2008) showing that Gly administered orally in rats decreases 5-HT and dopamine levels in the frontal cortex, midbrain and striatum. All these suggested that GBH induces depression and anxiety-like behavior observed in the treated groups.

On the other hand, the findings support a crucial role for perturbed amygdala 5-HT and enhanced glutamatergic mechanisms in the pathophysiology of mood disorders. Indeed, previous evidence supports the possibility that emotional disorders involve neuronal hyperexcitability in the amygdala (Tran et al., 2013). In support, Tran et al. (2013) showed that 5-HT depletion leads to the hyperexcitability of the central amygdala as the output of the nucleus, elucidating the anxiety-like behavior. Moreover, findings indicate that the depletion of 5-HT causes ventral mPFC neuron stimulation that excites principal BLA neurons elucidating anxiety phenotype (Russchen, 1982; McDonald, 1998). According to these findings, our results show that obvious serotoninergic circuitry disruptions observed in the DRN, the BLA and the ventral mPFC could underlie some structural and functional alterations potentiating the hyperexicitability of the amygdala and promote anxiety-like behavior.

While the mechanism underlying GBH-induced TH and 5-HT immunoreactivity reduction is not evaluated in the present study, it appears to be largely mediated by reactive oxygen species. As Gly can act as protonophore increasing mitochondrial membrane permeability to protons and Ca^2+^ (Olorunsogo, 1990), it can trigger the production of reactive oxygen species resulting in oxidative stress (de Liz Oliveira Cavalli et al., 2013). In human cell lines, both Gly and Roundup increased necrosis and apoptosis (Gasnier et al., 2009; Mesnage et al., 2013). Similarly, in rats, treatment with Gly and Roundup generated oxidative stress, induced lipid peroxidation (Astiz et al., 2009; El-Shenawy, 2009) and thus affected the oxidation-antioxidation homeostasis, promoting apoptosis and cellular death (Shimada et al., 1998; Yang and Sun, 1998), thereby inducing the anxiety and a depression-like phenotype and reducing motor development in the animals exposed to GBH.

In conclusion, we report for the first time the neurotoxic effect of subchronic and chronic subtoxic Roundup^®^ exposure in mice. Overall the results reported in the present study indicate that, as previously described for other OPs, GBH exposure during the juvenile and adult period leads to an alteration in the activity level of animals and their affective performances paralleled by the dopaminergic and serotoninergic system impairment. These results support the epidemiological findings that pesticide-exposed populations are susceptible to neurobehavioral changes.

## Author Contributions

YAB, SB-M and MB designed the experiments; performed the analysis of the data; YAB and MB performed the experiments; assembled the figures. All authors wrote or edited and validated the manuscript.

## Conflict of Interest Statement

The authors declare that the research was conducted in the absence of any commercial or financial relationships that could be construed as a potential conflict of interest.
